# Evaluation of Twitter data for an emerging crisis: an application to the first wave of COVID-19 in the UK

**DOI:** 10.1038/s41598-021-98396-9

**Published:** 2021-09-24

**Authors:** I Kit Cheng, Johannes Heyl, Nisha Lad, Gabriel Facini, Zara Grout

**Affiliations:** 1 Department of Physics and Astronomy, University College London, Gower Street, London, WC1E 6BT UK; 2 Department of Physics, University of Warwick, Coventry, CV4 7AL UK

**Keywords:** Computational science, Epidemiology, Applied mathematics

## Abstract

In the absence of nationwide mass testing for an emerging health crisis, alternative approaches could provide necessary information efficiently to aid policy makers and health bodies when dealing with a pandemic. The following work presents a methodology by which Twitter data surrounding the first wave of the COVID-19 pandemic in the UK is harvested and analysed using two main approaches. The first is an investigation into localized outbreak predictions by developing a prototype early-warning system using the distribution of total tweet volume. The temporal lag between the rises in the number of COVID-19 related tweets and officially reported deaths by Public Health England (PHE) is observed to be 6–27 days for various UK cities which matches the temporal lag values found in the literature. To better understand the topics of discussion and attitudes of people surrounding the pandemic, the second approach is an in-depth behavioural analysis assessing the public opinion and response to government policies such as the introduction of face-coverings. Using topic modelling, nine distinct topics are identified within the corpus of COVID-19 tweets, of which the themes ranged from retail to government bodies. Sentiment analysis on a subset of mask related tweets revealed sentiment spikes corresponding to major news and announcements. A Named Entity Recognition (NER) algorithm is trained and applied in a semi-supervised manner to recognise tweets containing location keywords within the unlabelled corpus and achieved a precision of 81.6%. Overall, these approaches allowed extraction of temporal trends relating to PHE case numbers, popular locations in relation to the use of face-coverings, and attitudes towards face-coverings, vaccines and the national ‘Test and Trace’ scheme.

## Introduction

Twitter as a popular social medium platform, is a host for users to express their opinions. Due to its abundance of textual data and user metadata, it can be used for the input of Machine Learning (ML) algorithms. Behavioural patterns and trends can be extracted from Twitter to give potential insights within society, and public attitudes can be better understood and predicted. These areas can be important in aiding government bodies through crisis management, particularly in exploring new methods to track the initial spread of a virus. Such examples that have been investigated include the use of wastewater testing^1^ and contact-tracing phone applications, as well as using social media data as a tool^2^. An unusual feature of COVID-19 is its asymptomatic nature, which had led to significant community transmission with symptoms being completely undetectable in individuals by using nationwide ‘Test and Trace’ schemes. This provides the necessary motivation to explore alternative approaches, particularly based on language processing, to predict the evolution of and attitudes towards the pandemic. This is of particular significance early in a pandemic when there is a significant strain on testing resources and equipment.

The focus of this study is on assessing the ability of Twitter as an outbreak predictor and behavioural analysis tool in the context of the COVID-19 pandemic, specifically during periods of emerging crisis before government intervention i.e. pre-mass testing. The aim was to investigate whether the total volume of tweets corresponding to a particular Twitter API query could be used as a proxy to model the evolution of PHE case numbers during the initial phase of an emerging global crisis and determine the time-delay for the occurrence of the next major outbreak. The ability to assess public opinion and correlate trends with reported news is also investigated during the initial outbreak using the textual content of tweets. The aim here was to determine the main subjects of discussion on Twitter surrounding the COVID-19 pandemic, as well as the prevalence of face-coverings in various locations in the UK. Face-coverings had been introduced by the UK government as a recommended precautionary measure initially, and later mandatory by law^3^, to help reduce the spread of the virus, and as such was a popular topic of online conversation.

The use of alternative methods to detect an initial global crisis outbreak, as well as the initial public response, can yield the necessary information for governments to respond effectively. The techniques and analysis discussed in this study can provide useful tools for early intervention of policy makers, particularly during periods of emerging global crisis before mass-testing is readily available.

## Material and methodology

### Data acquisition

A comprehensive COVID-19 Twitter data set containing all tweets from the UK over the duration of the pandemic would be ideal for performing outbreak prediction and behavioural analysis. However, due to limitations on the amount of data that could be collected via the Twitter API endpoints, a focused study on different cities in England was conducted. An initial exploratory analysis found that the cities with the largest fraction of tweets in the UK were London, Manchester, Liverpool and Birmingham, some of the largest cities by population in the UK.

In order to conduct regional analysis, the location of a Twitter user can be inferred in different ways. Typically tweet objects contain none, one or both of the following location-related attributes;^4^ location or geotagged. A geotagged tweet contains latitude and longitude coordinates tagged by the user enabling their precise location to be accessible. Whereas the location attribute is a user inputted free-form string, typically indicating a user’s home location on their profile. A preliminary analysis showed that approximately 2% of the tweet objects aggregated contained a non-null geotagged attribute. This provided a reliable source to determine regional dependence, however the volume of geotagged tweets obtained was insufficient to extract any meaningful trends. Approximately 97% of tweet objects were found to contain a non-null location attribute, where a user’s home location could be inferred. Non-essential travel in the UK stopped on March 16th 2020 alongside the first national lockdown which began on March 23rd 2020. This meant that the majority of the UK population were stationary to some degree in their daily movements. For this reason, a heuristic approach was taken where the location of a user was inferred via their profile location. More sophisticated methods would be needed in order to improve the reliability of user location in order to conduct further analysis on varying levels of location granularity, whether that be for the size of a town, city or county. An approach that may be useful in this setting could be to explore other methodologies whereby a user’s location could be inferred or extracted from their network of friends or the textual content of a subset of their tweets.

**Figure 1:**
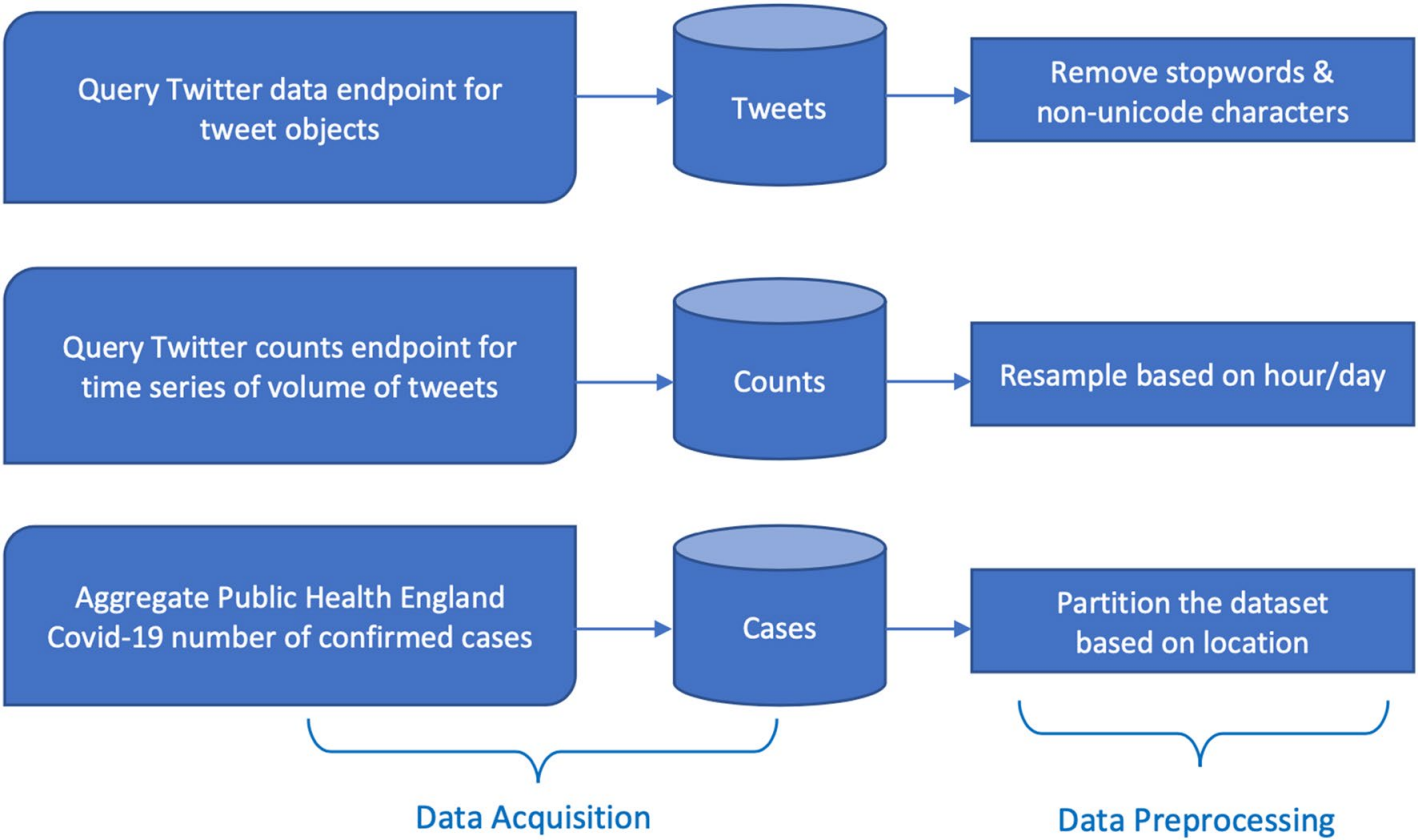
Workflow used to acquire, store and process the data obtained via the Twitter API data^8^ and counts^5^ endpoints, as well as the data obtained from Public Health England^7^ COVID-19 daily confirmed infection case numbers in the UK.

The workflow used to acquire, store and process the data collected from the Twitter API is shown in Fig. 1. The total volume of tweets matching a particular query can be retrieved per minute, hour or day via the counts endpoint^5^ of the Twitter API. This provides a time-series distribution of the frequency of a particular keyword(s) or hashtag(s) within a given location. Using online hashtag trackers^6^, it is found that the most frequently used hashtags and keywords over the course of the pandemic in the UK (February 1st - August 19th 2020) were #covid and #coronavirus, where hashtags are commonly used to give context and categorise the theme or common trends within a tweet. Symptom related keywords were also identified as high frequency use within tweets, such keywords included cough, fever and symptom. As such, queries were made against the Twitter API for tweets containing these hashtags and keywords, focusing on tweets located in the cities of London, Manchester, Liverpool, Birmingham and Leicester, between February 1st - August 19th 2020. These locations were chosen in order to investigate the first major peak in the outbreak in the four largest cities in the UK based on population size (London, Manchester, Liverpool & Birmingham). The city of Leicester was also chosen, which saw a significant second peak in case numbers during June 2020. It was of particular interest as it was one of the first cities in England to have a second wave at a time when community testing was being significantly ramped up. As such, there was an interest to see if this second outbreak could be observed in Twitter data. Tweets were also filtered to be of English language and retweets were negated. A similar approach was taken to determine the most popular keywords used in the context of face-coverings, where the most frequently used keywords were face mask and face covering. Table 1 shows the base queries used to build Twitter API queries for the counts endpoint. Daily confirmed infection case numbers recorded via PHE^7^ were also collected as a means to use in time-series analysis. Case numbers were partitioned based on the five UK locations of interest.

In order to build a corpus of tweets, the data endpoint^8^ of the Twitter API was used. This endpoint returns a maximum of 500 tweet objects per API request matching a particular query, where each tweet object contains the textual content of the tweet and user metadata. In order to sample the textual content of COVID-19 related tweets spanning the duration of the first major outbreak in the UK, the data endpoint was queried for UK originating tweets containing #covid OR #coronavirus OR covid OR coronavirus, as well as a separate query containing face mask OR face covering. The time period of the API query was structured to obtain a maximum of 500 tweets per hour between 09:00 - 22:00 GMT each day between 1st February - 19th August 2020. Table 2 shows the base queries used to build Twitter API queries against the data endpoint, where a corpus of $\sim$800,000 unique tweets were acquired.

**Table 1:** Base queries made against the Twitter API counts endpoint^5^ to obtain the total volume of tweets containing the above hashtags or keywords.

Base twitter API query
#Covid OR #coronavirus OR covid OR coronavirus lang:en -is:retweet profile_country:GB
‘Face mask’ OR ‘face covering’ lang:en -is:retweet profile_country:GB
Cough OR #cough lang:en -is:retweet profile_country:GB
Fever OR #fever lang:en -is:retweet profile_country:GB
Symptom OR #symptom lang:en -is:retweet profile_country:GB

Each query was made for the following UK locations: London, Manchester, Liverpool, Birmingham & Leicester, whereby hashtags and keywords were both queried. Aggregated tweets were filtered to be of English language and retweets were negated. The time period queried was between February 1st - August 19th 2020, spanning the duration of the first major peak in case numbers in the UK.

**Table 2:** Base queries made against the Twitter API data endpoint^8^ to obtain tweet objects containing the hashtags and keywords stated, where tweet locations originated from the UK.

Base twitter API query	Number of unique tweets
#Covid OR #coronavirus OR covid OR coronavirus	771,776
Face mask’ OR ‘face covering’	33,641

Aggregated tweets were filtered to be of English language and retweets were negated. The time period queried was between February 1st - August 19th 2020, spanning the duration of the first major peak in case numbers in the UK. The number of unique tweet objects obtained for each query during this time period is indicated.

### Time series analysis

In order to see how far ahead tweet volume was able to forecast an outbreak, the optimal time lag between the two had to be determined. Correlation analysis was performed by considering how the time series of the volume of tweets (extracted using the counts endpoint) for queries 1 and 3-5 in Table 1 correlated with the time series of deaths for each city under consideration. Spearman’s rank correlation coefficient was then used to assess any potential correlation. The time lag which maximised the correlation coefficient was deemed the optimal one.

### Topic modelling

To better understand the main themes of discussion within COVID-19 related tweets, which is not possible using tweet volume analysis alone, topic modelling was performed on the text field of the tweet objects. Topic modelling is an unsupervised text mining technique used to find patterns of word co-occurrence in a corpus. It can be applied within information retrieval, document classification and exploratory analysis of a large corpus. Latent Dirichlet Allocation (LDA) is an example of topic modelling which optimises the probability of a word belonging to a specific topic. It assumes that a corpus of text can be described by a distribution of topics, where each topic can be defined by a distribution of words^9^. The presence of each word in a document is thus assigned to a topic based on its probability, leading to a topic mixture for the document. This technique has been successfully applied in social science settings to analyse online support group posts to identify coherent topics^10^.

An LDA model was trained on the corpus of tweets using the Gensim Python library^11^ in Python. As tweets were not labelled by topic, there was no prior knowledge of how many topics may exist. Hence, these underlying topics within the corpus were said to be ‘latent’ or hidden. To initiate the model, the number of topics and the a-priori belief for each topics’ probability (usually inversely proportional to the number of topics) were specified. Nine topics with equal probability were chosen based on a quantitative topic coherence metric^11^ during hyperparameter tuning and interpretability of the resulting topic word clouds. There is a level of subjectivity in the final step which required manually assigning topic names to each word cloud.

### Named Entity Recognition (NER)

To further characterise the content in a tweet, a Named Entity Recognition (NER)^12^ model was used. NER models automatically find custom entities within a large corpus of unlabelled text, by utilizing a semi-supervised learning approach in order to learn the vector space of phrases to discriminate. Starter seeds are provided to the model to initialize the vector space, followed by iteratively manually labelling additional samples, retraining and correcting the model’s predictions as required.

The model was initially trained to recognise location-based entities from a pre-trained word vector model, extracted from a corpus of Reddit comments^13^. This formed the basis of the token-to-vector layer which contains vector representations for each multi-word expression. The aim was to train a model to recognise location-based entities within the context of tweets containing facemask or face covering and thus determine how the unique mentions of location keywords evolved over time. A list of seed keywords were then provided in order to direct the model close to the target vector space. These seeds included; ‘airport’, ‘bus’, ‘restaurant’, ‘pub’, ‘supermarket’. After training on labelled text, the model can assign a specific class to entities it recognises. New phrases and suggestions were outputted from the Reddit corpus with a high similarity score to the seeds. These entities, known as ‘match patterns’, together with a sample of 600 tweets, were manually annotated using the Prodigy annotation tool^14^ for the presence of location-based words forming a ground truth dataset. The NER model was then trained and applied on all ‘mask’ related tweets to identify the presence of a location.

### Sentiment analysis

A recent literature review presented the use of sentiment analysis based on opinion-lexicon methods, in order to analyse text sentiment, extract data from social media such as Twitter and the application of sentiment analysis to world events, healthcare, politics and business^15^. In this study, two methods were used to evaluate the time evolution of fraction of tweets with negative, neutral and positive sentiments related to specific topics.

The first method was the Valence Aware Dictionary for Sentiment Reasoning (VADER) model^16^. This text sentiment analysis model is sensitive to both polarity (positive or negative) and strength of emotion. VADER computes a normalised score (or ‘compound score’), from summing the valence score of each word in the input text. Here, valence is a measure of ‘goodness’ or ‘badness’ of a word which was determined from 10 independent human annotators. The compound score ranges from − 1 (extremely negative) to +1 (extremely positive). A threshold for ‘positive’ was set to be greater than 0.5, whereas ‘negative’ was set to be less than − 0.5 and anything in between was neutral. The output of VADER was validated qualitatively by examining the word clouds of the words in a tweet being classified as negative, neutral, and positive. This was one of the benefits of using VADER as the contribution of each word to the sentiment of the tweet was known. The large number of ‘neutral’ tweets was largely due to the choice of thresholds used for the compound score. A narrower threshold for neutral sentiment would produce less neutral tweets, and more of either negative or positive. Since the compound score is a sum of valence scores of each word and normalised to between − 1 and 1, it is possible that positive and negative words cancel out leading to a compound score closer to 0 thus neutral.

As a comparison to the out-of-box VADER sentiment analyser, a random forest classifier was trained on the Sentiment140 dataset^17^, which has found widespread usage in analysing the sentiment in tweets^18^. As the Sentiment140 dataset does not contain any neutral tweets, it was assumed that tweets for which the classifier was equally unsure about the sentiment were neutral. As such, a softmax output was used to predict the probabilities of the two classes. The difference between these probabilities was taken, giving a range between – 1 and 1. Any tweet with a “net sentiment” between – 0.5 and 0.5 was assumed to be neutral, as this implied that the classifier could not firmly classify the sentiment as either positive or negative.

Any differences in the fractions of tweets of varying sentiment can be attributed to the datasets that informed the outputs of the respective tools. VADER is rule-based and based on a valence measure, whereas the random forest classifier was trained on a labelled dataset. As such, each classifier will assign different words with different value for the sentiment, hence resulting in sentiment spikes at different times. However, in order to better inform the sentiment analysis, it would be more suitable to train a classifier on a more topically relevant labelled dataset in order to better capture the features that give tweets about specific topics a certain sentiment. However, this approach was not taken due to time constraints of the project. As such, the main drawback with both methods is that it may be inaccurate for domain specific terms. For example, ‘positive case’ would have a positive sentiment despite having a negative connotation in the context of the pandemic. Similarly, linguistic-subjective phenomena such as irony and sarcasm may also be misinterpreted.

## Results and discussion

### Initial outbreak detection

The use of social media data to look at the prevalence of a disease has been considered in the past for avian influenza^19,20^, and has also been considered for coronavirus^2,21,22^. This phenomenon of Twitter’s ability to provide insights into pandemic activity has been referred to as “wisdom of the crowds”; the collective knowledge of individual users^2^. By making use of this collective knowledge, the objective is to try and predict pandemic activity in the absence of any virus tracking system. In order to build an early-warning system, the total volume of tweets obtained via the counts endpoint was utilised within a time-series comparison with Public Health England (PHE) datasets on COVID-19 deaths^7^. Using deaths as a means of tracking the outbreak was more meaningful than using confirmed COVID-19 cases. The reason for this is the limited testing capacity in the UK at the onset of the pandemic, which meant that the number of positive tests would only ever have been an underestimate of the true number. The number of deaths is a more reliable number. The following sections discuss the time-delays determined by correlating both time-series and an evaluation to what extent Twitter can be employed as an outbreak predictor.

**Figure 2:**
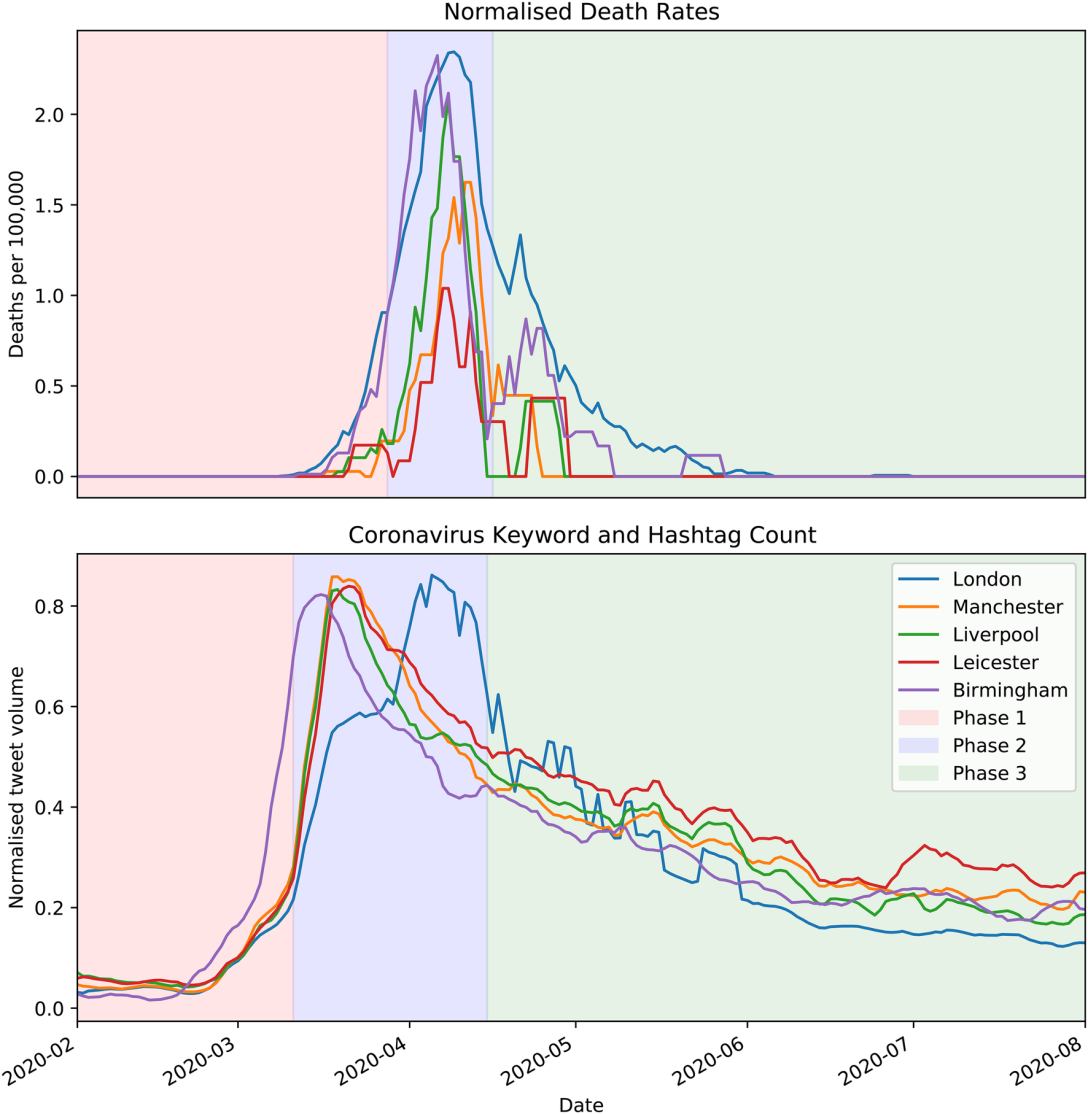
Top: Positive UK COVID-19 death numbers per 100,000 plotted as a function of time, reported via Public Health England UK government data^7^. Bottom: The total number of tweets containing the keyword ‘coronavirus’, aggregated via the counts endpoint via the Twitter API. Both distributions exhibit three phases, Phase 1: an initial exponential rise, Phase 2: the peak in the time series and Phase 3: a gradual decay.

The number of positive deaths per 100,000 was extracted from the PHE dataset for London, Manchester, Liverpool, Birmingham and Leicester. Figure 2 depicts the time series of the deaths per 100,000 for these five cities, as well as the total number of tweets aggregated per day matching the query #covid OR #coronavirus and covid OR coronavirus. Both distributions evolve with three distinct phases. The first phase is an initial exponential rise, the second phase depicts reaching a maximum and the third phase is a gradual decay. Despite a much-publicised second wave in Leicester, there was no second wave in the city’s COVID-19 deaths. A second wave of new infections was found from PHE data, but this could also be attributed to more widespread community testing.

For all cities under consideration, it was observed that the total volume of tweets precedes the case numbers. This factor could prove to be extremely useful in initial disease tracking stages, as was the case with Ebola in some regions of Nigeria^23^. For the Coronavirus, many governments struggled to identify cases swiftly due to the asymptomatic nature of the disease, as well as the fact that its symptoms made it hard to distinguish from other ailments. For this reason, it makes sense to make a distinction between an “informal outbreak”, when the number of tweets on the subject increase exponentially, and a “formal outbreak”, which is when the death numbers exhibit the same feature. A similar approach has been adopted in a previous publication^22^.

The correlation between Twitter query counts specified in Table 1 and PHE death numbers was considered. In each case, the aim was to determine the lag which maximises the correlation coefficient between the two time series. The strength of the correlation is determined through comparison with the null hypothesis, such that the two time series are uncorrelated. This procedure was repeated for the five UK locations.

**Figure 3:**
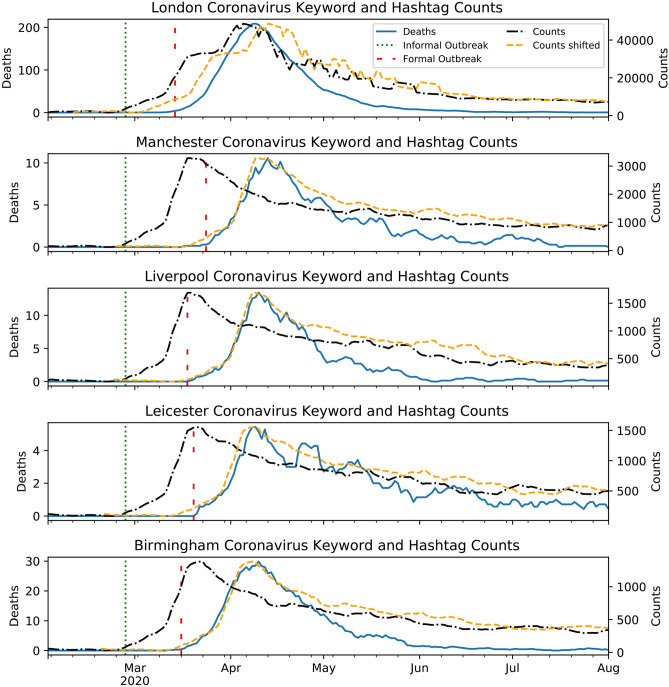
Plots of the time series of the counts endpoint for ‘coronavirus’ or ‘#coronavirus’ or ‘covid’ or ‘#covid’ for five UK cities as well as death numbers between February and August 2020. Also plotted is the former shifted by the time lag that maximised the correlation coefficient. The dates of the formal and informal outbreaks are plotted as red and green vertical lines, respectively.

Table 3 summarises the lag-time determined for each UK location which maximised the correlation coefficients for the time series of the query in question correlated with the PHE death numbers. The correlation was determined using the Spearman Rho coefficient. For all cities, the correlation coefficients are found to be in the range $0.7-$ 0.95, this corresponds to rejecting the null hypothesis at the $p<0.01$ level. From these results, it is clear that the query: (#covid OR #coronavirus) and (covid OR coronavirus) provides the earliest indication of an outbreak, on average. Figure 3 shows plots of the time series of the counts for the (#covid OR #coronavirus) and (covid OR coronavirus) query as well as the coronavirus case numbers. The figure also shows the shifted Twitter counts time series that maximises the correlation coefficient.

**Table 3:** Summary of lags determined between Twitter query counts^5^ time series and PHE death numbers^7^ between February 1st - August 19th 2020. A positive lag of *x* days indicates that the Twitter query time series precedes the PHE deaths by *x* number of days.

Base twitter API query	Lag (days)				
	London	Manchester	Liverpool	Birmingham	Leicester
(# Covid OR #coronavirus) and (covid OR coronavirus)	6	22	21	17	17
Cough OR #cough	20	27	23	20	20
Fever OR #fever	11	22	19	15	21
Symptom OR #symptom	8	22	21	17	18

This method has shown success in predicting the onset of the first peak in case numbers in the UK cities considered. The optimal lags for the cities are comparable to the ranges found for the coronavirus using Google Trends^21,24^ as well as Twitter^2^. The aforementioned works all considered the spread of the virus across entire countries, as opposed to localised levels. This gives further validation for a time lag of 6-27 days for an outbreak to develop. This range of lags matches values found previously for the US^22^. All of this suggests that the “wisdom of the crowds” in predicting a virus outbreak is independent of the geographical size or location of the region.

Finally, it should be noted that this method should by no means be relied on as the only means of outbreak prediction for either the coronavirus or any other future epidemic. The keywords and hashtags used to track the evolution of Twitter discussions related to COVID-19 during the period of February 1st - August 19th 2020 may evolve over time and no longer be relevant throughout the course of the pandemic. Further work would be needed to investigate trends in hashtag/keyword usage over time, particularly in response to new government measures and pandemic-related news, i.e. a second lockdown and vaccine trials. Additionally, when considering smaller populations, one important factor to take into account may be geographical nesting with larger hubs in the region, as it is possible that there will be a significant amount of commuting between these locations. This would contribute to the spread of the virus as well as provide a greater number of Twitter counts^2^. An example of geographical nesting would be Leicester and Leicestershire, which have large numbers of people commuting between them. Considering the case numbers of each in isolation would not provide an accurate picture of the outbreak. Leicester was also different to the other UK cities considered, as it had a highly-publicised second wave due to an outbreak in a factory. It was noted in another work that considered symptom-related searches^25^ that as more clinical manifestations of the coronavirus are noted and published, it is likely that the keywords and hashtags used in tweets will change. If one were to try to use specific keywords as indicators of localised outbreaks, one would need to be tracking the top hashtags as a function of time to identify relevant topics being discussed by users.

Furthermore, it might be difficult to compare lag values for different countries. This is due to the fact that countries made use of different non-pharmaceutical interventions (NPIs). A combination of the fact that the nature of the NPI would have differed as well as the timing would mean that lags found for different countries or regions may not always be directly comparable. It has been found that both of these factors have a significant effect on the evolution of the pandemic^26^. However, the UK initially imposed a uniform national lockdown, which for the time period considered meant that different cities could be meaningfully compared. It should be noted that comparisons between cities would not prove as meaningful for the UK for the period of time in which a tiered lockdown system was used.

### Public interest, behaviour and sentiment evaluation

The use of social media posts could provide vital information about popular opinions surrounding heavily discussed topics such as COVID-19 and has the potential to gauge the adoption of certain policies such as face masks in public. While the time series analysis can provide an indication of when and where an outbreak might happen, it ultimately only considers gross numbers. In order to get a better idea of how people are responding to the pandemic and any NPIs, the textual content of the tweets must be analysed. The following sections describe the use of various natural language processing techniques to explore the COVID-19 related content on Twitter.

#### Topic modelling

**Figure 4:**
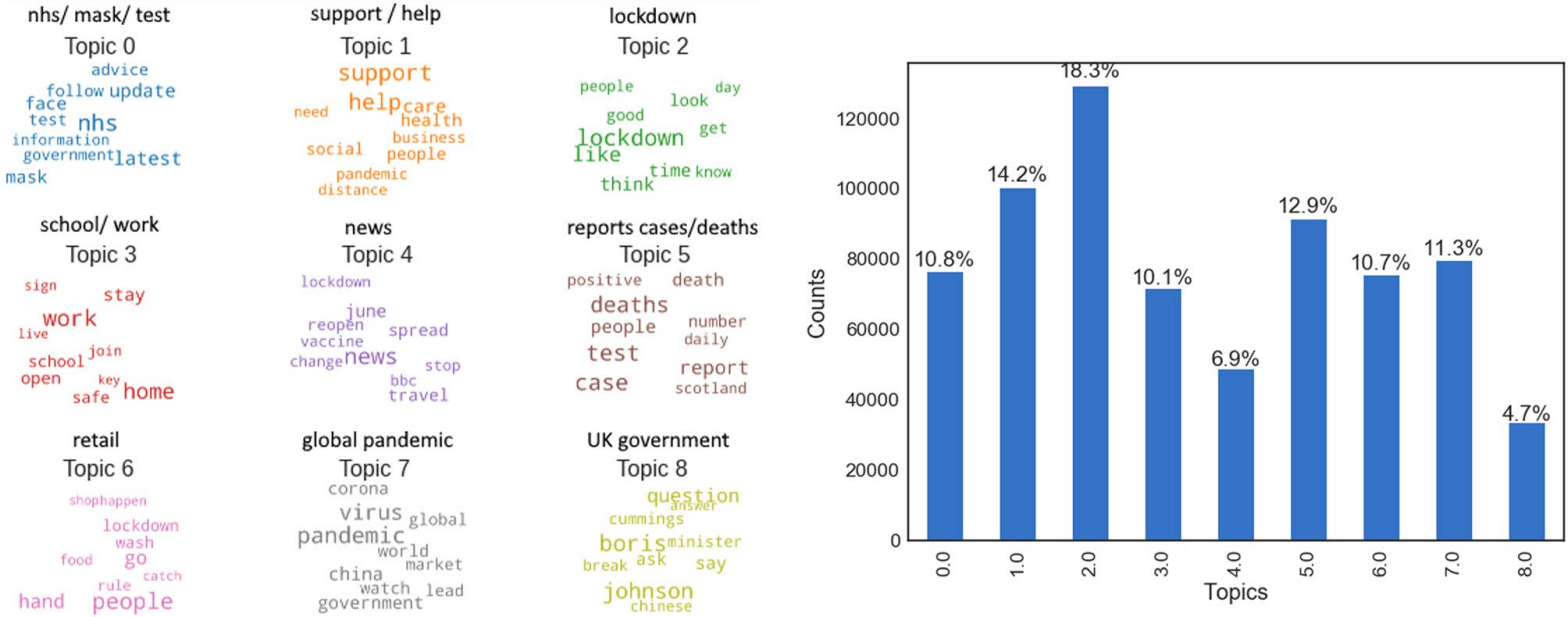
Left: topic word clouds derived from Latent Dirichlet Allocation (LDA) model topic estimation. Right: The number of tweets from each topic, whereby a tweet is assigned the topic with the highest probability.

The LDA topic model was applied onto the entire corpus of tweets with nine initial topics. The algorithm produced nine sets of words, one for each of the hidden topics. For each topic, the set of words were interpreted to a theme: ‘nhs’, ‘support/help’, ‘lockdown’, ‘school/work’, ‘news’, ‘reports cases/deaths’, ‘retail’, ‘global pandemic’ and ‘UK government’. The top ten words for each topic are shown as word clouds in Fig. 4 (left), the larger the word, the more weight it has in defining the topic. The topic model appears to have learned the underlying topics well based on the qualitative coherence of the word groups. The topics were examined in more detail for a sample of tweets. For simplicity, the topic with maximum probability was assigned to each tweet. The most discussed topic in this corpus of tweets was ‘lockdown’ reflecting the enormous public interest at the time (shown in Fig. 4 (right)). This is an expected result given the timeliness of data collection spanning the first UK lockdown which started on March 23, 2020^27^ with restrictions lasting into July, an extraordinary measure which many have not experienced before.

However, the results of this analysis should only be viewed as an overview of the main topics in the corpus, there could in fact be a greater number of underlying topics which were not captured given the pre-chosen number of topics. Using this unsupervised technique with an unlabelled corpus of tweets without truth information for topic label, proved challenging to assess the overall purity of the topics. However, this approach offered a heuristic overview of broad themes discussed within a large corpus such as tweets related to COVID-19.

#### Identifying locations associated with face coverings

The trained NER model learnt the vector space of match patterns containing location keywords in the training dataset and achieved a precision of 81.6%, recall of 77.6% and a combined F1 score of 79.5% on the test dataset (20% of annotated tweets). The advantage of this model is its ability to identify similar entities within this vector space without the need to hard code all possible examples of locations or different forms of the same location. This model was used to extract all tweets with location keywords. Another powerful feature of the NER model was its ability to identify n-grams which match the vector space it was trained on. The model classified 24% of tweets which mention a location ($\sim$8000 tweets) between April 1 to August 19, 2020.

Having improved the purity of location-related tweets, the time evolution of the mentioning of locations, as well as sentiment were evaluated. Using lemmatization, a list of unique locations was obtained, such examples include ‘shop’, ‘supermarket’, ‘public transport. By considering the cumulative frequency of each location term as a function of time, a bar chart race animation was produced showing how different location words change in popularity as time progresses. It was found that the top five locations mentioned were: ‘shop’, ‘public transport’, ‘public’, ‘supermarket’ and ‘bus’. It was also found that a significant portion of tweets ($\sim 5\%$) were from online businesses (e.g. Redbubble and Etsy) selling masks on Twitter contributing to almost four times more mention of the word ‘shop’ than the next most popular location ‘public transport’.

The workflow demonstrated here with the NER model to isolate relevant tweets from an unlabelled corpus is general and effective. For the purpose of this study, locations relating to face masks were of interest. Equally, this technique could be applied to other content of interest. For example, finding the most common COVID-19 symptoms people report on social media^28^, or finding tweets which indicate COVID-19 infection and COVID-19 awareness^29^. Other studies such as^30^ also demonstrated methods of discriminating tweets which reported infection from those that expressed concerned awareness of the flu. In their study, the authors used Amazon Mechanical Turk to label 11,900 tweets as concerned awareness, infection, media and unrelated, to train a classifier which yielded F1 scores of between 0.72 to 0.80. The NER methodology demonstrated in this study may have many further benefits, including reducing human resources and the time needed to produce a labelled ground truth.

#### Mask prevalence

It was found from social media posts that the adoption of face coverings varied across different locations. Using Twitter data, the prevalence of the use of face coverings in the UK was assessed in different locations such as shops, supermarkets and public transport .

The location NER model was applied on the dedicated ‘face covering’ dataset (see Table 2) to extract a subset of tweets relating to the wearing of face coverings at some location while removing tweets originating from sales advertisements. Tweets were filtered using the verb ‘wearing’ as it was the most frequently used verb in the dataset and the progressive form of the verb ensured that ‘wear’ was being used as an action, rather than as a noun or perhaps in another context. Applying this to the ‘face covering’ dataset, it was found that 3% of tweets ($\sim$1000 tweets) satisfied the requirements above. Each tweet was split at punctuation, and at conjunction words such as ‘if’ or ‘as’ which were used to connect two clauses to form one sentence. This isolated the sub-sentence which directly refers to the ‘wearing’ of face coverings. Finally, sub-sentences were then classified as a correct reference of ‘wearing’ face coverings, or a reference of ‘not wearing’, based on the presence of negative adverbs such as ‘not’, ‘barely’ and ‘only’, or negative nouns such as ‘no’ and ‘nobody’. This allowed satisfactory classification of tweets with an accuracy of 88% based on a test set of 100 examples. It was found that 83% of tweets mentioned people wearing masks. Table 4 shows a breakdown by location.

A similar analysis was executed to break down by geographical location. ‘Greater London’ and ‘City and Borough of Manchester’ were found to have more than four times the number of tweets compared to the next most frequent region likely linked to population size. Looking at the fraction of tweets for ‘wearing’/ ‘not wearing’, London was found to have the lowest percentage of ‘wearing’, but still relatively high at 77% (Table 5). It is worth noting that only approximately 72% of the tweets had ‘sub-region’ field populated. Due to only $\sim$1000 tweets satisfying the criteria of having a location mention and the verb ‘wearing’, behavioural change over time regarding the wearing of face coverings at locations was too sporadic to draw meaningful conclusions. Other attempts were made to enlarge this dataset such as filtering with the word ‘wear’ only. This relaxed filtering lead to lots of ‘noise’ in the dataset such as tweets promoting the use of face coverings, which does not reflect whether people are actually wearing a mask. Further work should consider identifying new rules to enlarge such a dataset to allow for a time-dependent behavioural analysis to be performed.

In England, the wearing of face coverings in enclosed public spaces became legally mandatory from July 24, 2020^31^, whereas these results show the sentiment and attitude towards face masks before they were introduced by the UK government. This prior knowledge would provide key insight into the behaviour of the general public and may help policy makers in future decision making.

**Table 4:** Percentage of tweets mentioning ‘wearing’ and ‘not wearing’ at different locations.

Location	Wearing (%)	Not wearing (%)	Count
Shop	81	19	261
Public	85	15	175
Public transport	74	26	72
Train	74	26	53
Bus	77	23	57
Supermarket	87	13	53

**Table 5:** Percentage of tweets mentioning ‘wearing’ and ‘not wearing’ at different geographical regions.

Sub-region	Wearing (%)	Not wearing (%)	Count
Greater London	77	23	226
City and Borough of Manchester	98	2	198
Borough of Oldham	100	0	45
Borough of Tameside	100	0	39
Essex	84	16	38

**Figure 5:**
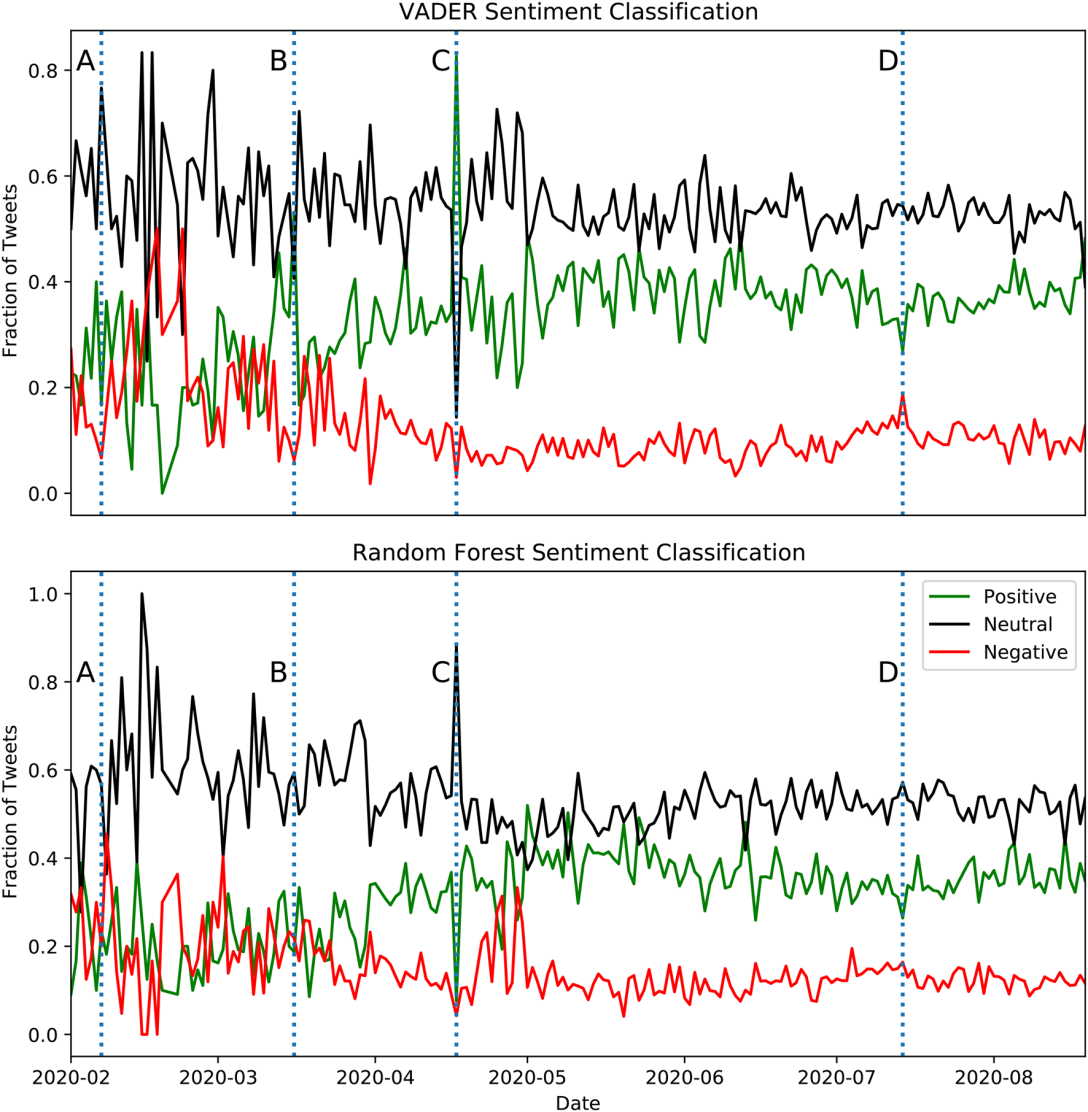
Time series of the three kinds of sentiment for the tweets about masks as predicted by VADER and a random forest classifier trained on the Sentiment140 dataset. We observe that the general trends are broadly similar, with roughly 40% of tweets being positive from May 2020 onwards. Four major events are marked on the time series. A corresponds to a WHO warning about a global mask shortage on February 7th, 2020, B corresponds to an announcement by the Health Secretary about high-quality masks being distributed to the NHS on March 16th, 2020, C corresponds to a government announcement that scientific advice would be followed regarding face masks on April 17th, 2020 and D corresponds to an announcement that governments would make face masks mandatory in shops in England on July 14th, 2020.

#### Sentiment analysis

Sentiment analysis on pandemic related topics such as ‘test and trace’, ‘vaccine’ and ‘facemasks’ was performed in order to gauge how the public feels about these rather controversial topics. Furthermore, the time evolution of the mask dataset was considered by what fraction of tweets were positive, negative or neutral per day. Two separate tools were used to classify the tweets in order to compare their results. Figure 5 shows how the fraction of tweets that had each sentiment changed as a function of time, as predicted by the two tools used.

**Figure 6:**
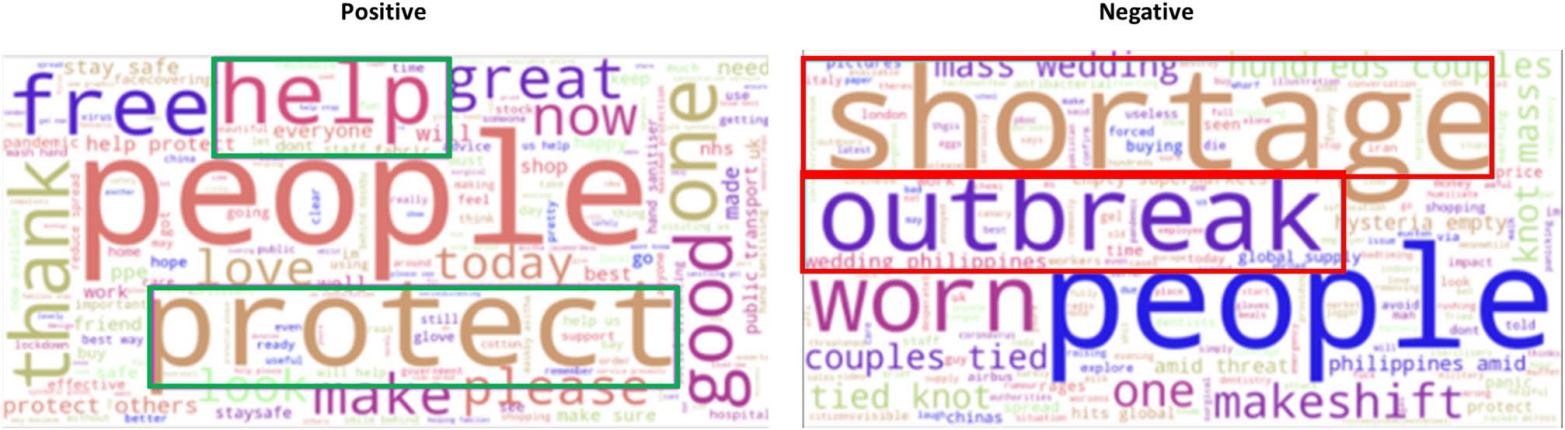
Word clouds of the corresponding positive (left) and negative (right) mask related tweets from Vader sentiment analysis. We observe rather distinct types of words in each, particularly more words of gratitude in the positive word cloud as expected.

For ‘masks’, people expressed positive sentiment overall. It was found that major news or announcements also corresponded to clear spikes in a particular sentiment. Figure 5 shows four labelled events. ‘A’ corresponds to a WHO warning about a global mask shortage on February 7th, 2020 which saw a period of high negativity between February and March. ‘B’ corresponds to an announcement by the Health Secretary about high-quality masks being distributed to the NHS on March 16th, 2020 which saw a positive sentiment spike, ‘C’ corresponds to a government announcement that scientific advice would be followed regarding face masks on April 17th, 2020 which saw another positive sentiment spike and ‘D’ corresponds to an announcement that governments would make face masks mandatory in shops in England on July 14th, 2020 which saw a negative sentiment spike. To illustrate that the tweets were discussing these particular topics, Fig. 6 shows that the negative tweets pre-March (in relation to ‘A’) were mostly concerned with the ‘shortage’ of masks amidst the inevitable outbreak in March^32^. On the other hand, a significant number of positive tweets expressed how masks can ‘help’, ‘protect’, and keep people ‘safe’.

For ‘test and trace’, an overall positive sentiment was found. However, there were significantly more negative tweets in April. These negative tweets were concerned with data protection like breaking GDPR laws^33^. In contrast, positive tweets supported the scheme saying that it would help to control the virus.

For ‘vaccine’, the sentiment was overall positive. However, there were significant numbers of negative tweets with terms like ‘Bill Gates’ and ‘Gates wants’, tying in with the conspiracies surrounding Bill Gates^34^. In contrast, the positive tweets were focused around the strong immune response of the Oxford vaccine and offering ‘hope’^35^.

It was also of interest to see whether there is a geographical north/south difference in sentiment towards facemasks. However, the analysis showed no significant difference. A major limitation was the small number of tweets with location information. Furthermore, there were about nine times less tweets from Northern England than Southern England, limiting the usefulness of the comparison.

Currently, sentiment analysis cannot distinguish between an angry tweet about someone who did not wear a mask in a shop versus a positive tweet about the need for people to wear masks to prevent the spread of the virus or about the policy in general. In both cases, the tweet suggests an individual and opinion which is pro-mask and pro-Govt policy. Further work should focus on intent analysis to estimate the change in compliance to specific government policies in relation to events, news, announcements, such as government officials breaking the rule of ‘stay at home’. This type of insight would be very useful for policy makers to see the effectiveness of their policies.

## Conclusions and outlook

The use of Twitter as a data medium for crisis management has been shown to provide key insights and behavioural patterns about a demographic. A variety of tools have been developed to analyse Twitter data during the period surrounding an emerging crisis when mass-testing is not in place. The results of this work can be summarized as follows.

By leveraging “wisdom of the crowds”^2^ in conjunction with the use of time series analysis, the distribution of total volume of tweets for a given query provides a good foundation to build a prototype early warning system, as well as the ability to identify potential hotspots. The selection of hashtags or phrases used within the Twitter query is crucially important due to the dynamic nature of language. It was found that increased usage of various coronavirus-related keywords was strongly correlated with a rise in COVID-19 deaths 6–27 days later.

Topic modelling was used to reveal nine distinct topics within the corpus of COVID-19 related tweets, including themes ranging from retail to government bodies. By utilising a Named Entity Recognition (NER) algorithm in a semi-supervised manner within the extracted topics, tweets containing location-based keywords within an unlabelled corpus were extracted with a precision of 81.6%. This combination of techniques not only allowed for further analysis of the textual content to be investigated, but also increased the purity of topic selection.

The use of an out-of-the-box sentiment analysis tool (VADER), as well as a random forest classifier trained on the labelled Sentiment140 dataset^17^ have worked well on extracting sentiment polarity from tweets with sentiment spikes corresponding to major news or announcements. This demonstrates the potential for policy makers to leverage sentiment analysis to evaluate public feeling for a policy.

Further work should consider the reliability of user location, the selection of dynamic hashtag queries , the ability to generalise the procedure to other countries, as well as extending the procedure to incorporate intent analysis for policy makers. Despite some limitations, the techniques investigated have the potential to help predict future global crises and evaluate public response both indirectly and efficiently.

## Data Availability

The datasets generated and analysed during the current study are available from the corresponding author on reasonable request.
